# Editorial: Application of natural bioactive compounds in animal nutrition

**DOI:** 10.3389/fvets.2023.1204490

**Published:** 2023-05-16

**Authors:** Balamuralikrishnan Balasubramanian, Wen-Chao Liu, In Ho Kim

**Affiliations:** ^1^Department of Food Science and Biotechnology, College of Life Science, Sejong University, Seoul, Republic of Korea; ^2^Department of Animal Science, College of Coastal Agricultural Sciences, Guangdong Ocean University, Zhanjiang, China; ^3^Department of Animal Resource and Science, Dankook University, Cheonan-si, Chungnam, Republic of Korea

**Keywords:** bioactive, antioxidants, microbiota, immunity, gastro-intestinal, growth, poultry, livestock

## Introduction

The current decade is facing an array of challenges in animal production such as global warming-induced heat stress, cost production in rearing and transportation, antibiotic-free meat production, encompassing the weanling time period, and mycotoxin contamination-stored animal feeds. Diminutive lacunae in the above domains can lead to serious animal health issues, which can cause acute physiological disorders and deteriorate the entire animal production percentage. They are also associated with health issues in consumers, which reflect greatly in the overall health status of stakeholders. Hence, there is a pressing need to address these problems and provide strategies to advance the animal production sector toward sustainability. The focus of this special issue inclined toward inviting ideas on the identification and utilization of natural bioactive compounds from microorganisms, plants, algae, and sea weeds, etc., for enhancing animal nutrition. Multi-drug antibiotic resistance has been an alarming issue in treating the diseases; however, the adoption of these bioactive metabolites can provide cues for disease treatment and tolerance in animals. A very low quantity of these bioactive metabolites has been reported to offer copious nutrient benefits and pharmacological effects. The intangible reasons of utilizing these compounds are due to lacunae in the scaling-up process and the inadequacy of insights in the mechanism of their action. Further, several bioactive compounds are still unexplored and elusive; thus, it is crucial to explore the interplay of these compounds in the health and production of farm animals. In addition, a targeted delivery of these compounds through inter-disciplinary aspects such as nanotechnology will bring about technological intervention in animal feed production and management strategies. Twenty articles based on the augmentation of bioactive compounds for the betterment of gut function, reduction of oxidative stress, decontamination agents of mycotoxin, and nano-encapsulation-based approaches for improvement in animal nutrition were received.

## Role of plant-based metabolites in improving the health status of poultry

Chronic exposure to heat stress (HS)-induced disorders in behavioral, immunological, metabolic, physiological, hormonal, and biochemical mechanisms lead to a decline in the average daily feed intake (ADFI), production of egg, efficiency of feed, and quality of eggs in poultry. Further, HS directly aggravates oxidative stress and disturbs the homeostasis of the antioxidant system. This oxidative stress impacts the internal organs, GI tract, liver, and production of egg yolk or albumen and blood. The dietary augmentation of curcumin and plant extract blend of *Scutellaria baicalensis* in the form of a phytonutrient solution (PHYTO) improved the quality of the young layers, improved the thermotolerance, and upregulated the antioxidant defense system in young layers (25–32 weeks of age) grown in naturally higher temperature circumstances. This enhanced the intestinal villosity and liver performance. Moreover, it induced a translational effect in egg production, with better oxidative stability and eggshell breaking force (Giannenas et al.). However, further studies are needed to ascertain the synergism with the GI-associated microbiota. Curcumin has also been utilized for improving the growth indices and controlling insect pests of livestock (Sureshbabu et al.). In a similar study, the efficacy of *Artemisia ordosica* alcohol extract (AOAE) in augmenting the growth, immune, and inflammatory response in diets was studied with lipopolysaccharide (LPS)-challenged broilers. In other words, dietary supplementation of AOAE enhanced the spleen index value, decreased the bursa index, and alleviated the levels of IL-1β, IL-2, IL-6, IgG, IgM, and IL-4 in serum and associated immune response organs in LPS-challenged broilers. The upregulation and mRNA expression of TLR4, MyD88, TRAF6, NF-κB p65, NF-κB p50, IL-1β, and IL-6 genes were noted. The downregulation of IκBα and PPARγ demonstrated improved immunity and constrained inflammation (Shi et al.). The potential antioxidative and antiinflammatory activities of *Antrodia cinnamomea* polysaccharide (ACP) were evaluated in slow-growing broilers stimulated by LPS. The ACP diet accelerated the population of beneficial cecal microbiota such as *Lactobacillus, Faecali bacterium*, and *Christensenellaceae*, which curbed liver damage and brought about the synergistic effects of the antioxidants in the restoration of beneficial microbiota (Ye et al.). The mulberry leaf extract (MLE)-supplemented feed enhanced aspartate transaminase (AST) activity, increased the levels of glutathione peroxidase (GSH-Px), intensified the color of the egg yolk, elevated high-density lipoprotein (HDL-C) and superoxide dismutase (SOD) activity in the serum, and boosted the strength of eggshell. Further, the upregulation of PPARα and SIRT1 and decreased expression of FASN and PPARγ were evident (Zhang B. et al). Feed formulation using honeysuckle extract (HE) was experimentally studied using geese models, in which the growth index, biochemical parameters, immune responses, and gut microbiota were evaluated. The abundance of Bacteroidetes was increased, while the Firmicutes population decreased in HE-fed diets; however, *Bacteroides barnesiae, Subdoligranulum variabile, Bacteroides plebeius*, and *Faecalibacterium prausnitzii* were notably dominant. Therefore, the addition of honeysuckle extract to the diet impacted the intestinal function and immune tolerance by intervening in the gut microbial composition (Li G. et al.). The effects of Chinese yam polysaccharides (CYP)-fortified diets on the immune function of broilers were also studied. The treated group exhibited a higher thymus index and increased levels of serum IgA, complement C3, C4, IGF-I, T3, T4, INS, GH, IL-2, IL-4, IL-6, and TNF-α in the CYP-fed group. An improved spleen index and serum IgM and IgG concentrations corresponded to elevated immune response in broilers (Deng et al.). Long-term exposure to mycotoxins exerts oxidative stress; poultry in particular are very sensitive to aflatoxins (AFB1) that are common in animal feed commodities. AFB1 is reported to cause stunned growth, immunosuppression, and hepatotoxicity; however, dietary supplementation of plant-based polyphenolic compounds such as Chinese gallnut tannic acid (TA) was reported to catalyze the antioxidant enzyme activity and lower the malondialdehyde content in poultry. Moreover, it prevented the decrease of villus height/crypt depth ratio and liver enlargement in AFB1-treated broilers (Xi et al.).

Quercetagetin (QG) as a dietary supplementation for broilers improved the nutrient digestibility, intestinal morphology, immunity, and antioxidant capacity. It increased the levels of IgG and C4 levels in the blood and elevated the total antioxidant capacity (T-AOC) in serum, jejunum mucosa, and ileum mucosa through the *Nrf2*/antioxidant response element (ARE) signaling pathway mediated by Keap1 (Wu et al.). Supplementation of dandelion tannins or isoflavones of soy bean positively impacted the growth index, serum biochemical indexes, and antioxidant rates and accelerated the cecal microbiota abundance and overall intestinal health of female Wenchang chickens (Li X. et al.). Apart from the plant sources, bioactive compounds from yeast derivatives-supplemented feeds have the potential to be used as antibiotics, immunogenic factors, pathogen inhibitors, and intestinal health and growth promoters in livestock and poultry (Patterson et al.). Physical attributes such as thermal manipulation (TM) during incubation promoted alterations in embryonic development, hepatic amino acid metabolism, and hatching results in layer-type chickens (Han et al.).

## Herbal supplementation to improve the overall growth and immune response in livestock and *in vivo* models

Tannic acid-chelated zinc (TAZ) supplementation in the diet ameliorated PEDV-induced changes in new-born piglets. Malondialdehyde levels in the plasma duodenum, jejunum, and colon notably decreased; in parallel, TAZ relieved the oxidative stress and PEDV-induced damage in intestinal mucosa. Further, absorptive function and growth in piglets was improved, which suggested that TAZ can be a potential feed supplement for neonatal and weaning piglets (Zhang Z. et al.). The high-fat diet induced obesity and affected the liver function, and the chronic HF diet also induced megalohepatia, steatosis, inflammation, and hepatocyte apoptosis in rats. The chronic effects of grape seed proanthocyanidin (GSPE) in the liver function and lipid metabolic parameters were evaluated in rat models. The results advocated that GSPE stimulated the expression of the Wnt3a/β-catenin signaling pathway, which prevented endoplasmic stress and apoptosis in hepatocytes. In addition, microRNA-103 mediated a critical role in the signal transduction pathways. Therefore, it was suggestive that GSPE demonstrated a protective outcome on the liver and offered cues for value addition in animal feed. Further, GSPE downregulated the expression of the genes responsible for lipid synthesis, which prevented fat deposition in the liver (Sun et al.). The microbiota in the GI tract of livestock play a significant role in maintaining the intestinal health and the associated digestive processes. The hydrogen sequestration capacity of the archaea has been utilized in feed formulations to reduce methane emission and energy loss. Supplementation of green additive *Scutellaria baicalensis* and *Lonicera japonica* mixed extracts (SLE) in wheat bran improved the lactation, immunity, and production performance of piglets through a transmission effect. Elevated levels of glucose (GLU), triglyceride (TG), total cholesterol (TC), prolactin (PRL), and interleukin-10 (IL-10) were noted. Fat, IgA, and IgG in colostrum significantly increased post-supplementation in SLE diets (Wang L. et al.). *Lactobacillus plantarum* and *Pediococcus acidilactici* fermented feeds along with the basal diet enhanced weight gain, fecal acetate, and butyrate; moreover, it increased the abundance of short-chain fatty acid (SCFA)-producing microbiota in the gut of nursery pigs (Yang et al.).

## Role of metabolomics and molecular signatures in profiling of GI microbiota

The fortification of pet feeds with polyphenol-rich pre-biotic fiber blend improved the microbial saccharolytic and post-biotic metabolism, improved the stool quality, and decreased digestion-related disorders in pet dogs. Further, it was found to be effective in the early stages of the feed routine and to aid in the management of gastroenteritis (Fritsch et al.). The elaborative microbiome and metabolomic profiling threw light on the contribution of dietary fiber in maintaining the adult dog gastrointestinal health status.

Archaea species such as *Methano massiliicoccales* in beef cattle, *Methano brevibacter* in sheep, and *Methanobrevibacter smithii* have been reported to be involved in feed utilization. In context with the above cues, the composition and expression activity of the gut archaea were analyzed on a metagenomic and transcriptomic platform. The archaeal population was reported to be relatively low in the digestive tract; however, their transcript levels were extremely high. Hence, it was suggestive that the archaea were functional and active in regulating the GI health in monogastric animals (Peng et al.). The liver, the largest solid organ, performs vital metabolic functions and serves as a hub of several pathophysiological mechanisms. Understanding the non-coding RNAs (lncRNAs) and their roles in the lipid metabolism during growth and development was studied using rabbit models. The variations of lncRNA and mRNA transcriptomes in different stages of rabbit, from birth to somatic maturity, was evaluated using the RNA-seq bioinformatics platform. As a result, 38 differentially expressed mRNAs such as ACSS2 and 215 lncRNAs such as MSTRG.30424.1 were identified, which can be used as biomarkers to understand the vital roles of the lipid metabolism (Wang G. et al.). The impact of fucoidan on the intestinal health, colon morphology, and cecal contents of weaned lambs was investigated. Apparently, fucoidan improved the levels of propionic acid and butyric acid and elevated the antioxidant levels and immunogenicity, while 16S rDNA screening showed increased levels of beneficial intestinal bacterial strains. Further, fucoidan decreased the diarrhea rate, which relaxed the weaning stress. Moreover, fucoidan as milk replacer supplementation improved the large intestinal health of weaned lambs (Guo et al.). One of the hallmarks of plant-based compounds is that they relieve oxidative stress due to the presence of tannins, phenolics, and many other compounds. These can be well utilized as a cofactor in augmenting diet-based formulations for pets and laboratory model poultry and livestock . However, to attain a clear understanding and investigation of their antioxidant mechanism, clinical and experimental trials must be thoroughly performed. The scaling-up of these dietary supplements with industry–institution connections, technology transfer, patentable ideas, and commercialization would rightly benefit stakeholders and promote sustainable livestock production.

## Author contributions

All authors listed have made a substantial, direct, and intellectual contribution to the work and approved it for publication.

